# Deciphering Microbiome Related to Rusty Roots of *Panax ginseng* and Evaluation of Antagonists Against Pathogenic *Ilyonectria*

**DOI:** 10.3389/fmicb.2019.01350

**Published:** 2019-06-18

**Authors:** Defei Liu, Huanjun Sun, Hongwu Ma

**Affiliations:** ^1^Key Laboratory of Systems Microbial Biotechnology, Tianjin Institute of Industrial Biotechnology, Chinese Academy of Sciences, Tianjin, China; ^2^University of Chinese Academy of Sciences, Beijing, China

**Keywords:** biocontrol, *Ilyonectria*, *Panax ginseng*, rusty roots, microbiome

## Abstract

Plant roots host diverse microbes that are closely associated with root fitness. Currently, the relationship between microbes and rusty roots of *Panax ginseng* remains unclear. Here, we described the root-associated microbiome in rusty and healthy ginseng by metagenomic sequencing of 16S rRNA and ITS regions. Being enriched in Diseased-roots (Dr) of ginseng and their rhizosphere soil, the fungus of *Ilyonectria*, was identified as the most probable cause of the disease after ITS analysis. Meanwhile, an increase of *Mortierella* was observed in Healthy-roots (Hr). Surprisingly, an enriched *Fusarium* was found in both Hr and their rhizosphere soil. Besides, in comparison with Hr, decreased relative abundance of *Actinomycetales* and increased relative abundance of *Pseudomonadales* was observed in Dr after 16S rRNA analysis. What’s more, we isolated several microorganisms as antagonists that showed strong inhibiting effects on *Ilyonectria* in plate assays. In field trials, inoculation of *Bacillus* sp. S-11 displayed apparent suppression effect against *Ilyonectria* and shifted microbial communities in rhizosphere soil. Our research identified key microbiota involved in rusty roots of *P. ginseng* and offered potential biocontrol solutions to rusty disease.

## Introduction

Terrestrial plants are colonized by diverse microorganisms which include commensals, symbionts, and opportunistic pathogens (Muller et al., 2016). The colonized microbes present in endosphere and rhizosphere have a direct relationship with the fitness of plant roots. With recent advances in metagenomic sequencing and computational analysis, it is now possible to unravel root-associated microbiome (Lebeis et al., 2015; Castrillo et al., 2017). A previous study elucidated the microbes in disease-suppressive soils against pathogen in sugar beet (Mendes et al., 2011). They found several key bacterial taxa and genes involved in suppression of *Rhizoctonia solani*. Besides, a recent study revealed that *Flavobacteriia* was enriched in tomato rhizosphere, which was resistant to the soil-borne pathogen *Ralstonia solanacearum*. Pot trials showed that the inoculation of *Flavobacteria* had a beneficial role in defending *R. solanacearum* (Kwak et al., 2018). Therefore, metagenomics is a powerful tool for revealing the microbiota related to pathogen-triggered root diseases, offering possible solutions to preventing pathogen-infection in plants.

*Panax ginseng* Meyer (*Araliaceae*) has been an important herb of traditional medicine for thousands of years in East Asia. Mainly cultivated in China, Korea and Japan (Yun, 2001), *P. ginseng* is considered as an important cash crop due to its pharmaceutical properties contributed by ginseng saponins (Attele et al., 1999; Shin et al., 2015). As a kind of herbaceous perennial plant, it takes at least 5–6 years for ginseng to reach a marketable size. For those cultivated in mountain forest, more than 10 years is needed to grow to a good shape.

During continuous cultivation, there is a greater chance for ginseng to suffer from soil-borne diseases that lead to dramatic yield losses and quality decline. Major root diseases include root rots and rusty roots (Farh et al., 2018). The latter disease, also known as rusted roots or “the rust,” is characterized by small or large reddish-brown spots on the surface of ginseng roots (Supplementary Figure S1). It is widely reported in China, South Korea and Canada as a major cause of ginseng root deterioration at all stages (Hildebrand, 1934; Lee et al., 2011; Lu et al., 2015). Given the fact that ginseng roots are commercially graded according to their sizes, shapes and overall appearances, rusty root disease severely limits the output and quality of ginseng worldwide.

Presently, the causes of *P. ginseng* rusty roots are still controversial. It is mentioned in some studies that there was a close relationship between rusty roots and rhizosphere soil properties (Liu et al., 2014). Besides, microorganisms also play a key role in this disease as suggested in other studies that ginseng roots could be infected by putative pathogens, including both bacteria (Choi et al., 2005; Lee et al., 2011) and fungi (Rahman and Punja, 2005; Reeleder et al., 2006; Lu et al., 2015, 2019), and showed rusty symptom after inoculation of the isolates. However, there is no systematic research on the microbiome involved in the disease. Therefore, it is necessary to study the links between microbiome and rusty roots with metagenomic sequencing.

We used 16S rRNA and ITS sequencing to identify endophytic and rhizospheric microbiome involved in rusty roots of *P. ginseng*. After that, microbiota diversity and structure of healthy and rusty ginseng roots were evaluated. Moreover, pure-culture was used to isolate antagonists and their biocontrol abilities were assessed in pot and field trials. We also investigated the changes of microbial community in rhizosphere soil after inoculation of antagonists by metagenomic sequencing in field trials.

## Materials and Methods

### Sample Processing

Ginseng roots were sampled from Tonghua and Baishan, Jilin Province, China in August 2016 and July 2017. Twelve sites (2 m × 2 m) with different rusty root indices were selected, and 5–7 ginseng roots randomly collected from each site. Root samples were kept in a sterile plastic bag and transported to the laboratory in cold chain. The detail information of sampling sites was described in Supplementary Table S1.

Ginseng organs above the ground were aseptically removed and loose soil was physically removed until only soil adhered to the root surface remained. After that, loose soil was air-dried and crushed to pass through 2 mm nylon sieve to test the chemical properties. Soil pH was determined with a digital pH-meter (FE20-K, Mettler-Toledo, Switzerland) in a suspension of 1:2.5 soil/water ratio (w/v). Soil organic matter (SOM), available nitrogen (N), available phosphorus (P), and available potassium (K) were determined by using commercial chemical assay kits (Suzhou Comin Biotechnology Co., Ltd., China), respectively, according to the instructions. Soil chemical properties are shown in Supplementary Table S2.

The soil adhered to the root surface was processed according to the method of Lebeis et al. (2015). The roots were placed in a clean and sterile 50 mL conical tube containing 25 mL of phosphate buffer (6.33 g of NaH_2_PO_4_⋅H_2_O, 16.5 g of Na_2_HPO_4_⋅H_2_O, and 200 μL Silwet L-77 in 1 L of water). Rhizosphere soils were separated from the roots by vortexing the root system in buffer at the maximal speed (3000 rpm) for approximately 15 s. The turbid obtained was filtered through a sterile 100 μm nylon mesh cell strainer (Biologix Group Limited) into another sterile 50 mL conical tube to remove plant materials, sand, and other large debris. The filtrate was centrifuged in two steps to form a tight pellet and defined as rhizosphere soil sample. The rhizosphere soils from the same sampling site were mixed as one sample.

The rusty root indices of soil were calculated as followed:

(1) $$\mathrm{Rusty root index}=\frac{\Sigma \;(n\times \mathrm{rusty root grade})}{N\times \mathrm{the higest rusty root grade}}$$

where *n* equals to the number of ginsengs with different rusty root grades, *N* equals to the number of total ginsengs. Rusty-root grades range from 0 to 4, with 0 standing for healthy ginseng roots without rust, 1 for rust areas <10%, 2 for those covering 10–25%, 3 for those covering 25–50%, and 4 for those >50%. According to the rusty root index, we sorted out soil samples with index ≤0.5 as the Healthy-soil (Hs) group. While rest of the samples were classified as the Diseased-soil (Ds) group.

To obtain endophytic microbial communities, 3–5 roots from each site were selected according to the symptoms on the surface of ginseng roots. Roots were subsequently placed in new sterile phosphate buffer for sonication to remove soil and microbial aggregates left on the root surface by using an ultrasonic cleaner set on low frequency for 5 min (30 s bursts followed by 30 s rests). After that roots were washed again with sterile water. Then, Healthy-root (Hr) samples and Diseased-root (Dr) samples were excised (about 1–2 cm long by 0.5–1 cm wide and about 1 mm deep) from main-roots of ginseng with a sterile scalpel. For healthy roots without rusty spots and early lesion roots with small rusty parts, one piece of Hr sample was excised per root from asymptomatic parts. For those rusty ones, one Dr sample was taken per root from rusty parts. The details of samples were described in Supplementary Table S3.

Soil and root samples were flash frozen and stored at −80°C until DNA extraction with the Qiagen DNeasy PowerSoil Kit (Qiagen, Germany) following the manufacture’s protocol. For the root samples, we performed a pre-homogenization step using liquid nitrogen grinding.

### Pyrosequencing of the 16S rRNA and ITS Genes

The libraries were constructed and sequenced based on Illumina Miseq PE300 platform (Illumina, United States) according to the manufacturer’s protocols. Three sets of primers were used to amplify different regions of genes. For soil samples, the amplification of V3-V4 region of the 16S rRNA gene was achieved by using 338F and 806R. As for root samples, we amplified V5-V7 region of the 16S rRNA gene with 799F and 1193R, which displayed very low amplification of non-target DNA, such as plastid (mostly chloroplast) DNA and mitochondrial DNA (Beckers et al., 2016). While ITS1 and ITS2 were used concerning ITS sequence for both soil and root samples. The primer sequences were listed in Supplementary Table S4 and sequence data were deposited in GenBank (accession number: PRJNA512054).

### Bioinformatics Analysis of 16S rRNA and ITS Gene Sequences and Data Statistic Analysis

The 16S rRNA and ITS gene sequences generated were processed through the open-source software pipeline QIIME2 (Version 2018.4) (Caporaso et al., 2010). Quality control was performed using DADA2 plug-in for quality filtering, chimera removal and feature table generation (Callahan et al., 2016). As for data sequenced at different time, we used “qiime feature-table merge” to combine the results. Raw data sequence and quality filtering parameters were shown in Supplementary Table S5. The command of training feature “qiime feature-classifier” using Greengenes (McDonald et al., 2012) and UNITE (QIIME release, Version 01.12.2017^1^) as reference was utilized to classify representative sequences from our datasets. Features of 16S rRNA which were assigned as “Chloroplast” and those of ITS with no taxonomic assignment at kingdom level (27.9% for root samples and 0.3% for soil samples) were subsequently removed from the datasets.

Alpha-diversity, represented by Shannon index and Pielou’s evenness, was calculated using QIIME2 pipeline based on the feature table. Before the calculation of alpha-diversity, samples were rarefied to the same sequence depth (Supplementary Table S6), which also applied to beta-diversity calculation. Principal coordinate analysis (PCoA) was performed on the basis of Bray-Curtis distance matrix calculated in QIIME2 and visualized by EMPeror (Vázquez-Baeza et al., 2013). Permutational multivariate analysis of variance (PERMANOVA) (Anderson, 2001) with 999 permutations was used to detect statistical significances in QIIME2.

In this work, we presented linear discriminant analysis (LDA) effect size (LEfSe) to perform differentially abundant taxa between healthy and rusty groups (Segata et al., 2011). Redundancy analysis (RDA) was performed via Canoco for Windows 5 (Microcomputer Power, NY, United States).

Alpha-diversity indices and relative abundance of abundant taxa were tested with methods described below. Those of root and soil samples were tested by Student’s *t*-test using R (version 3.5.0), while those of biocontrol treatments were tested using one-way ANOVA followed by LSD *post hoc* test by IBM SPSS Statistics 19 (SPSS Inc.).

### Isolation of Microorganisms

Rusty root tissues were used in attempts to isolate causal agents. To begin with, roots were washed again with sterile water, and sections of rusty tissues were cut off. Root pieces were surface-sterilized by being first immersed in 1% sodium hypochlorite for 30 s, then in 70% ethanol for 30 s and finally rinsed in sterile water for three times. After that, disinfected periderm tissues (about 1 mm deep) were torn off with a pair of sterilized forceps and cut into small pieces (0.5 mm × 0.5 mm). Then the pieces were placed on a plate with potato dextrose agar (PDA) (6 g potato dextrose, 20 g glucose, 18 g agar, and 1 L distilled water) amended with 50 μg/mL tetracycline. Then, inoculated PDA plates were kept in darkness at 22 ± 1°C. After 7 to 14 days, fungal hyphae appeared. Pieces of hyphae were excised with an inoculating needle, placed on a new PDA plate and incubated at 22 ± 1°C.

After that, we isolated antagonists against fungal pathogens from ginseng root, Hs and uncultivated soil. First, asymptomatic sections from healthy roots or regions of apparently healthy tissue on diseased roots were conducted as described above. Three to five disinfected periderm pieces were placed separately on nutrient agar (NA) plates (3 g beef extract, 5 g peptone, 5 g NaCl, 18 g agar, 1 L distilled water, and pH 7) for bacteria isolation, Gauze’s No. 1 agar plates (20 g soluble starch, 1 g KNO_3_, 0.5 g K_2_HPO_4_, 0.5 g MgSO_4_⋅7H_2_O, 0.5 g NaCl, 0.01 g FeSO_4_⋅7H_2_O, 18 g agar, 1 L distilled water, and pH = 7.4 – 7.6) for actinomycete isolation and PDA plates for fungi. Apart from antagonists from root, soil microbes that might inhibit pathogens were also isolated. Serial dilutions were prepared up to 10^−4^ using sterile phosphate-buffered saline (PBS). Next, 100 μL of each diluted sample was spread onto plates with NA, Gauze’s No. 1 agar medium and PDA, respectively. Plates with NA and Gauze’s No. 1 agar were incubated at 30 ± 1°C for 7 days. While plates with PDA were incubated at 22 ± 1°C for 7 days. Bacteria and actinomycete colonies were transferred to new NA and Gauze’s No. 1 plate and purified by streak plate.

Identification of the purified isolates was achieved using multilocus sequence analysis. Firstly, Total genomic DNA was extracted from bacteria colonies or fungi fresh mycelia by using bacterial genomic DNA extraction kit (BioTeke Corporation, China, Cat#: D3350-01) and rapid fungi genomic DNA isolation Kit (Sangon Biotech, China, Cat#: B518229-0100), respectively. Afterward, PCR was performed in 50 μL reaction mixture containing 1 μL of DNA, 2 μL forward primer (10 mM), 2 μL reverse primer (10 mM), 25 μL 2 × Taq Plus MasterMix (Dye) (CWbio Corporation, China, Cat#: CW2849M), and 20 μL sterile PCR-grade water. Bacterial 16S rRNA genes were amplified by PCR with the primer pair of 27F and 1492R (Lane, 1991) for an initial denaturation at 94°C for 2 min, following by 30 cycles of 94°C for 30 s, 56°C for 1 min, 72°C for 1 min, and a final elongation step of 72°C for 10 min. As for *GyrB* gene of *Bacillus* and *Streptomyces*, primers UP1 and UP2R (Yamamoto and Harayama, 1995) and gyrBPF and gyrBPR (Guo et al., 2008) were used, respectively. The amplification was performed under the amplification conditions described in references. In the case of ITS gene of fungi, we used primers ITS5 and ITS4 (White et al., 1990) with the following modifications to the amplification protocol: 35 cycles, annealing at 52°C for 30 s. We also amplified intergenic spacer (IGS) sequences of fungi by PCR using the primers LR12R and invSR1R (Supplementary Table S4) with modifications to protocol: 35 cycles, annealing at 52°C for 1 min. Then the PCR amplified products were separated by agarose gel electrophoresis and sequenced at Genewiz (Tianjin, China). Bacteria were identified on the basis of similarities to 16S rRNA and *gyrB* sequences while fungi identification according to ITS and IGS sequences with their morphological characteristics. The sequences of the isolates were searched in NCBI^2^ and have been deposited in GenBank under the accession MK459368, MK459369, MK512096, MK512097, MK459418-MK459425, and MK512088-MK512095.

### Pathogenicity Test

To confirm the pathogenicity of isolated pathogens, a test was carried out via bare-root inoculation as described by Reeleder et al. (2006) with some modifications. Healthy ginseng roots (3-year-old) were washed with tap water and root surfaces were sterilized as described. *Ilyonectria robusta* (*I. robusta*) 4D-1 (4D-1) and *I. mors-panacis* TH5 (TH5) were randomly selected from pathogen isolates obtained from rusty ginseng roots. 4D-1 and TH5 were grown on PDA for 14 days at 22 ± 1°C. The endoconidia were washed and adjusted to 1 × 10^7^ endoconidia/mL. Sterilized filter paper strips (about 1 cm wide by 8 cm long) were immersed in endoconidia suspension for 30 s. Meanwhile, paper strips immersed in sterile water for 30 s were used as control. Then, treated paper strips were placed on the top and middle parts of the roots (4 replicates for each treatment). The inoculated parts in the middle were wounded with a sterile scalpel (about 1 cm long and 1 mm deep). Then, every inoculated root was wrapped with four layers sterile moistened gauze and placed in a folded plastic bag. Bags were incubated at 22 ± 1°C in dark for 3 weeks. After that, pathogens were recovered from symptomatic roots and confirmed by analyzing the ITS sequence.

### *In vitro* Inhibition of *Ilyonectria*

Microorganisms isolated from both soil and healthy ginseng roots were evaluated for their activities against *Ilyonectria*. In order to do that, PDA disks (5-mm diameter) containing 14-day-old 4D-1 mycelia were placed at the center of PDA plates. While isolates with possible antagonism were placed 2.5 cm away from them (3 replicates per isolate). Three weeks after inoculation, putative suppressive isolates were selected based on the width of the inhibition zone or inhibition rate. The activities of selected isolates against TH5 were also evaluated.

### *In vivo* Evaluation of Antagonistic Microbes

The pathogen 4D-1 was grown in potato dextrose broth (PDB) for 7 d at 22 ± 1°C, while three kinds of antagonisms were cultivated, respectively. For *Bacillus* sp. S-11 (S-11), it was cultured in nutrient broth (NB) for 36 h at 37 ± 1°C. Besides, *Streptomyces* sp. S6-31 (S6-31) was cultured in Gauze’s No. 1 broth for 5 days at 30 ± 1°C. As for *Trichoderma koningiopsis* S7-1 (S7-1), it was grown in PDB for 7 days at 25 ± 1°C. After that, concentrations of 4D-1 and S7-1 were observed with a hemocytometer, while concentrations of S-11 and S6-31 were determined by flat colony counting. Concentrations of all isolates were adjusted before use.

Biocontrol activity was determined in pot experiments with five treatments: CK: sterilized water; TI: 4D-1 (1 × 10^5^ endoconidia/g of dry soil); TB: S-11 (1 × 10^6^ CFU/g of dry soil) with 4D-1 (1 × 10^5^ endoconidia/g of dry soil); TS: S6-31 (1 × 10^5^ CFU/g of dry soil) with 4D-1 (1 × 10^5^ endoconidia/g of dry soil); and TT: S7-1 (1 × 10^5^ endoconidia/g of dry soil) with 4D-1 (1 × 10^5^ endoconidia/g of dry soil). All treatments were arranged according to the principle of randomized complete blocks. Each treatment contained five pots with each pot serving as a replicate. Every pot was planted with 4 1-year- old ginsengs and placed in a greenhouse at 22 ± 2°C with 14 h of sunlight (without direct sunlight) and 10 h darkness. Soil water content ranging from 30 to 50%. During our experiments, root colonization of inocula was assessed at 0, 10, 20, 30, and 40 days after inoculation. Every time, three roots were sampled randomly in each treatment for the extraction of genomic genes of rhizosphere soil as described above for qPCR analysis. Forty days later, incidence and severity of the disease was evaluated.

Biocontrol studies were also conducted in field with 4-year-old ginsengs severely impacted by rusty disease in Jilin province. During our trial, four treatments were applied by root watering: CK: sterilized water; TB: S-11 (1 × 10^6^ CFU/mL); TS: S6-31 (1 × 10^5^ CFU/mL); and TT: S7-1 (1 × 10^5^ endoconidia/mL). Each treatment consisted of three replicates arranged in a randomized complete block design. Each block consists of about 50 plants. Five roots were sampled randomly in each block and mixed as one sample at 40 days after inoculation. Then root colonization of isolates and biocontrol activities against *Ilyonectria* were evaluated by qPCR. The effects of inoculum on soil microbiome were also evaluated by pyrosequencing of 16S rRNA gene and ITS gene.

### Real-Time qPCR

A quantitative real-time PCR (qPCR) assay based on standard curves that used C*_t_* value was developed for the detection and quantification of *I. robusta*, *I. mors-panacis*, S-11, S6-31, S7-1, and genus of *Ilyonectria*. In order to develop a standard curve for target genes, we first amplified target fragments cloned in PMD-19 Simple T vector (Takara, Japan) with primers listed in Supplementary Table S4. Then, concentration of linear recombinant plasmids was determined by NanoDrop 2000 spectrophotometer (Thermo Scientific, MA, United States).

All PCRs were performed by Faststart Universal SYBR Green Master (ROX) kit (Roche, Switzerland) in LightCycler 96 System (Roche Diagnostics) with reaction volume being 25 μL. Each reaction contains 1-fold FastStart Universal SYBR green Master, PCR-grade water, 150 nM forward and reverse primer, and 2.5 μL soil DNA extract. After preparation, PCRs were operated as followed: denaturation for 10 min at 95°C followed by 40 cycles of denaturation for 15 s at 95°C, annealing and extension at 60°C for 60 s. Then melting curve analysis at the temperature ranging from 60°C to 95°C at the rate of 0.2°C/s was conducted after amplification.

## Results

### Metagenomic Sequence Analysis

When preparing for sequencing, root samples with low DNA concentrations or qualities after PCR were discarded. After quality filtering, 766 561 high-quality reads of total 16S rRNA V5-V7 sequences from 31 root samples were obtained. As for fungi sequencing, 736 894 high-quality ITS reads from 36 root samples were acquired. We also sequenced bacterial 16S rRNA V3-V4 and fungal ITS sequences of 12 ginseng rhizosphere soil samples, thus obtaining 260 541 and 406 810 high-quality reads, respectively. Concerning soil samples treated with inoculation in field trials, we obtained a total of 227 322 and 358 510 high-quality sequences for 16S rDNA V3-V4 and ITS sequences, respectively.

### Microbiota Differences Between Healthy and Rusty Ginseng Roots

**FIGURE 1:**
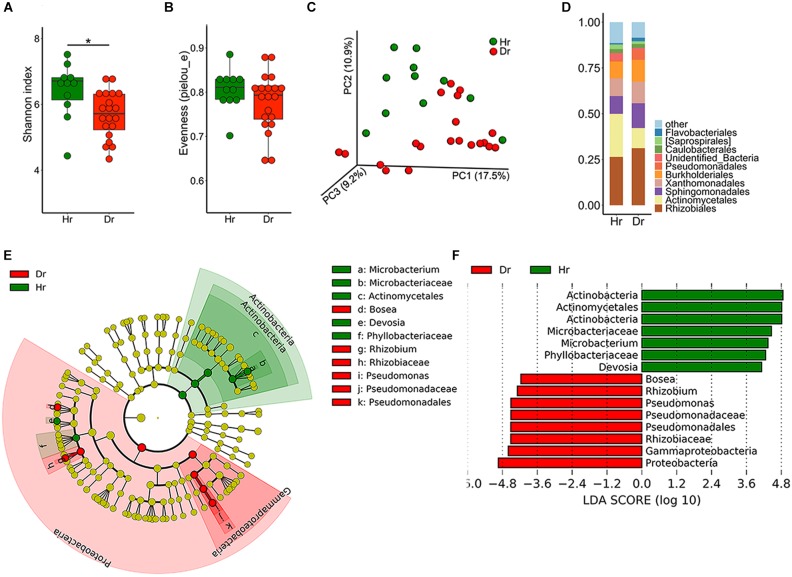
Bacterial microbiota composition of Healthy-root (Hr) and Diseased-root (Dr). Boxplot of alpha-diversity indices for 16S rRNA of Hr and Dr: **(A)** Shannon index; **(B)** Evenness (pielou_e) (Group significant indicated by asterisks: ^∗^*p* < 0.05). **(C)** Principal coordinate analysis (PCoA) of Bray-Curtis distances of the bacterial communities in Hr and Dr samples. **(D)** Relative abundances of bacterial orders in Hr and Dr samples. **(E)** Cladogram generated from LEfSe analysis, showing the most differentially abundant taxa enriched in microbiota from Hr or Dr. **(F)** LDA scores of the differentially abundant taxa shown in **(E)**. Taxa enriched in bacterial microbiota from Hr or Dr are indicated with a positive or negative LDA score, respectively (taxa with relative abundance >0.001 and LDA score >4 are shown).

To elucidate the distribution and assembly patterns of microbial communities related to rusty ginseng roots, a high-throughput sequencing approach was used. Compared with the Hr group, Dr had a significantly decreased bacterial Shannon diversity (Figure 1A), and no significant difference was detected in evenness (pielou_e) index (Figure 1B), which meant that Dr had a lower species richness. The dissimilarity between samples was explored using PCoA and the result revealed that the bacterial compositions of the two groups distinct with each other (Figure 1C, *F* = 2.185, *p* = 0.002). After sequence annotation, we found that *Pseudomonadales* increased from 1.03 to 5.15% (*t*-test, *p* = 0.02) (Figure 1D) among the identified orders in Dr group, whereas *Actinomycetales* decreased from 23.5 to 10.0% (*t*-test, *p* = 3.402 × 10^−4^). Moreover, on order level, LDA effect size (LEfSe) indicated that *Pseudomonadales* enriched in Dr while *Actinomycetales* enriched in Hr (Figure 1E,F).

**FIGURE 2:**
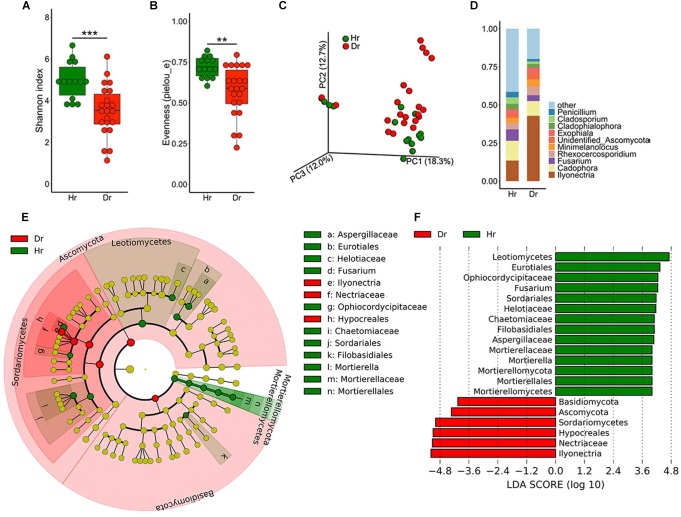
Fungal microbiota composition of Healthy-root (Hr) and Diseased-root (Dr). Boxplot of alpha-diversity indices for ITS of Hr and Dr: **(A)** Shannon index; **(B)** Evenness (pielou_e) (Group significant indicated by asterisks: ^∗∗^*p* < 0.01, ^∗∗∗^*p* < 0.001). **(C)** PCoA of Bray-Curtis distances of the fungal communities in Hr and Dr root samples. **(D)** Relative abundances of fungal genera in Hr and Dr samples. **(E)** Cladogram generated from LEfSe analysis, showing the most differentially abundant taxa enriched in microbiota from Hr or Dr. **(F)** LDA scores of the differentially abundant taxa shown in **(E)**. Taxa enriched in fungal microbiota from Hr or Dr are indicated with a positive or negative LDA score, respectively (taxa with relative abundance >0.001 and LDA score >4 are shown).

As for fungal community diversity in root samples, a marked drop in fungal alpha-diversity was observed in Dr group (Figure 2A,B), which revealed Dr group had decreased species richness and evenness. Meanwhile, PCoA result demonstrated dramatic discrepancies in the structure of fungal community (Figure 2C, *F* = 2.227, *p* = 0.005) between groups of Hr and Dr. After assignment, we found that the fungal microbiome showed severe dysbiosis in rusty roots. On genus level, some genera of fungi predominated (30–90%) in these samples. The predominated fungi were mainly (71%) assigned as the genus of *Ilyonectria*. Compared with Hr, obviously higher relative abundance of *Ilyonectria* was observed (*t*-test, *p* = 5.924 × 10^−4^) (Figure 2D). LEfSe results showed that the genera of *Ilyonectria* and *Basidiomycota* gathered (LDA > 4) in Dr while the fungal genera enriched (LDA > 4) in Hr were *Mortierella* and *Fusarium* (Figure 2E,F).

### Microbiota Differences Between Rhizosphere Soil of Healthy and Rusty Ginseng Roots

We also studied the diversity of microbiome in ginseng rhizosphere soil (Supplementary Figure S2). After analysis, no significant differences were detected in Shannon diversity or evenness (pielou_e) between Hs and Ds (Supplementary Figures S2A,B,E,F). For bacteria, LEfSe results revealed that Ellin309 and *Xanthomonadales* enriched in Ds while *Alteromonadales* enriched in Hs on the level of order (LDA > 2, Supplementary Figure S2D). On the other side, ITS sequencing results showed that *Ilyonectria* took up 0.02% of the relative abundance in Hs on genera level. While that percentage in Ds was 15.1%, presenting a significant difference (*t*-test, *p* = 2.145 × 10^−3^) when compared with Hs. This result was also confirmed by LEfSe (LDA > 2) (Supplementary Figure S2H). Besides, an enrichment of *Fusarium* was observed in Hs.

**FIGURE 3:**
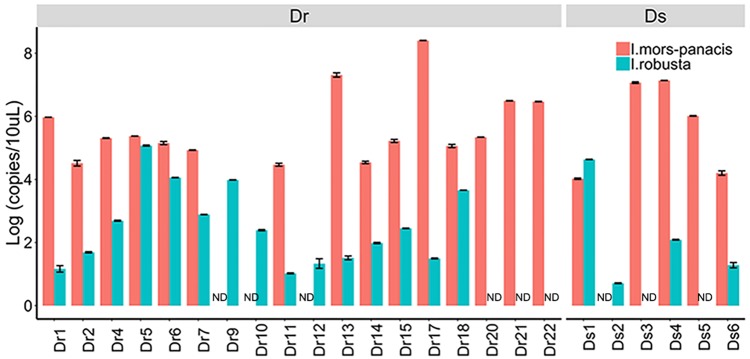
Quantification of *I. mors-panacis* and *I. robusta* in Diseased-root (Dr) samples and in Diseased-soils (Ds) (ND: not detected).

After comparison, we found that *Ilyonectria*, as a genus of fungi colonizing in ginseng roots, was the most abundant fungus in Dr and enriched in Dr and Ds as revealed by LEfSe analysis. In the following experiments, we isolated *I. robusta* and *I. mors-panacis* from rusty roots and the pathogenicity was confirmed by bare-root inoculation (Supplementary Figure S3). In order to figure out the dominant specie, the qPCR approach was used to detect copies of *I. robusta* and *I. mors-panacis* in rusty ginseng roots (relative abundance of *Ilyonectria* >29%) and their rhizosphere soil (Figure 3). The results showed that both *I. robusta* and *I. mors-panacis* were found in 12 rusty samples and three soil samples. While in the remaining samples, either *I. robusta* or *I. mors-panacis* was observed. Thus, we speculated that both the two species contributed to rusty disease. Based on these results, we conclude that the infection of *Ilyonectria* from rhizosphere soil is very likely to be the predominant cause of the rusty disease.

### Relationship Between Soil Physicochemical Properties and Microbial Abundances

**FIGURE 4:**
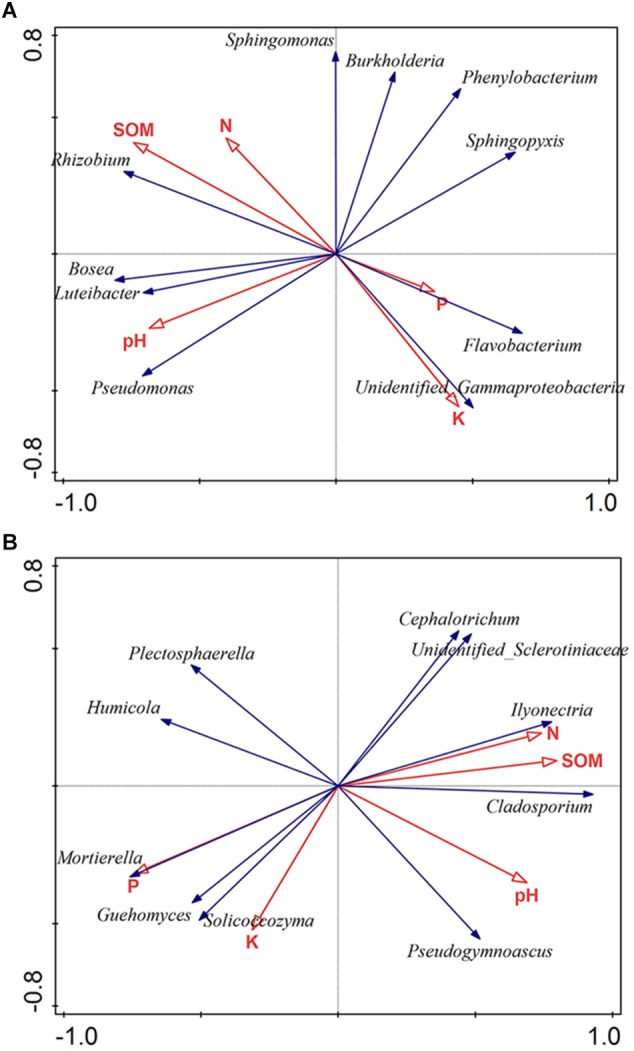
Redundancy analysis (RDA) of the bacterial **(A)** and fungal genera **(B)** corresponding to soil properties. The most 10 best fitting genera were shown (relative abundance >0.001). SOM: soil organic matter; N: available nitrogen; P: available phosphorus; and K: available potassium.

Soil characteristics have a great influence on soil microbial communities. To clarify the relationships between microbiota and soil properties, we used RDA with SOM, N, P, K, and pH being environmental variables (Figure 4). The most 10 best fitting genera were shown (relative abundance >0.001). The total explanatory variables account for 57.8 and 71.9% of bacterial and fungal variations, respectively.

Soil pH was considered as a dominant factor of soil bacterial communities (Girvan et al., 2003). According to our findings, pH plays a crucial part in shaping the microbiota composition of bacteria orders (Monte-Carlo test, *p* < 0.05). Also, the bacterial community was strongly affected by SOM (Monte-Carlo test, *p* < 0.05) (Figure 4A). Regarding to fungi, the result showed that fungal community was obviously impacted by P (Monte-Carlo test, *p* < 0.05), N (Monte-Carlo test, *p* < 0.05), SOM (Monte-Carlo test, *p* < 0.05), and pH (Monte-Carlo test, *p* < 0.05). Among fungal genera, *Ilyonectria* was positively related with SOM, N and pH, but negatively related to P (Figure 4B).

### Biocontrol Activity Evaluation of Antagonists and Root Colonization

In order to find a biocontrol strategy against *Ilyonectria*, pure-culture was used to isolate pathogens and antagonists. Four *I. robusta* and 3 *I. mors-panacis* from rusty roots were obtained as possible pathogens, while 205 bacteria and 82 fungi were isolated from ginseng roots and soils as potential antagonists for biocontrol activity experiments. Among all isolates, 11 bacteria and 12 fungi showed antagonistic activities and three of them showed strong inhibiting effects on 4D-1 and TH5 on PDA plates (Supplementary Figure S4), namely *Bacillus* sp. S-11 (S-11), *Streptomyces* sp. S6-31 (S6-31) and *T. koningiopsis* S7-1 (S7-1).

**FIGURE 5:**
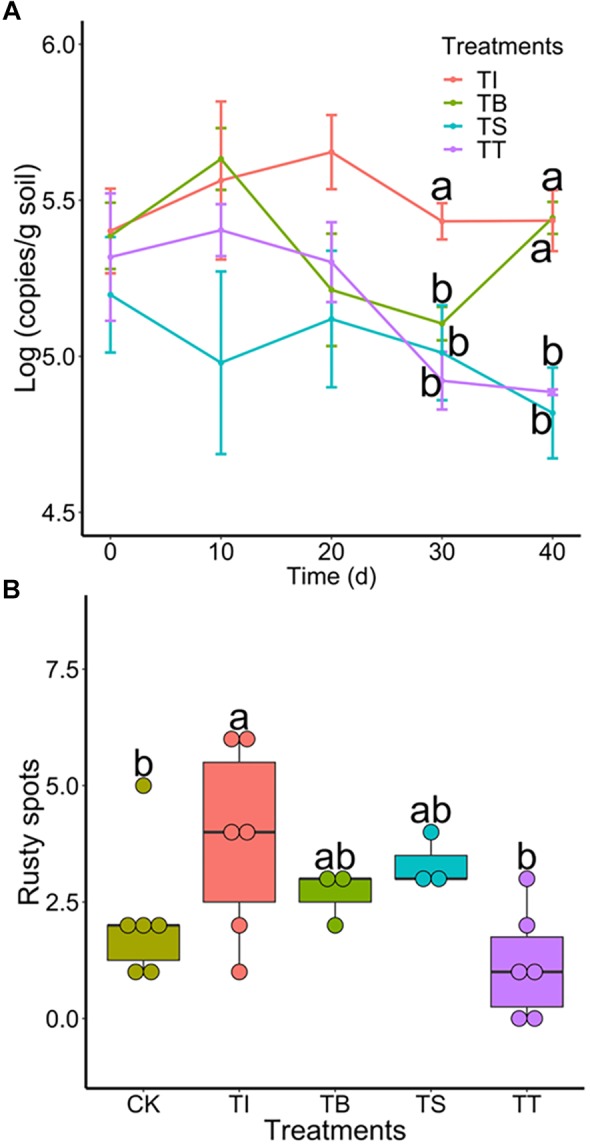
**(A)** Population dynamics of *I. robusta* 4D-1 in rhizosphere soil under pot trial (TI: 4D-1; TB: S-11 + 4D-1; TS: S6-31+ 4D-1; and TT: T S7-1+4D-1). **(B)** Rusty root disease severity was evaluated by the number of rusty spots (diameter >2 mm) on the skin of ginseng roots under pot trial (CK: control; TI: 4D-1; TB: S-11 + 4D-1; TS: S6-31+ 4D-1; and TT: S7-1+4D-1).

To investigate the colonization of antagonists and their suppression of *Ilyonectria* in rhizosphere soil under pot experiments, we tracked population dynamics of antagonistic microbes and 4D-1 by qPCR. The results (Supplementary Figure S5) showed that S-11 and S7-1 increased on day 10 and a population density above 3.86 × 10^3^ and 1.50 × 10^4^ (copies/g soil) was maintained until day 40, respectively, while S6-31 declined all the way along. Meanwhile, all antagonists showed antifungal activities in the soil with 4D-1 being significantly suppressed on day 30 or 40 (Figure 5A). Besides, we counted rusty spots on ginseng root skin on day 40 as the indicator of rusty disease and the result showed that treatment TT had the least rusty spots (Figure 5B).

**FIGURE 6:**
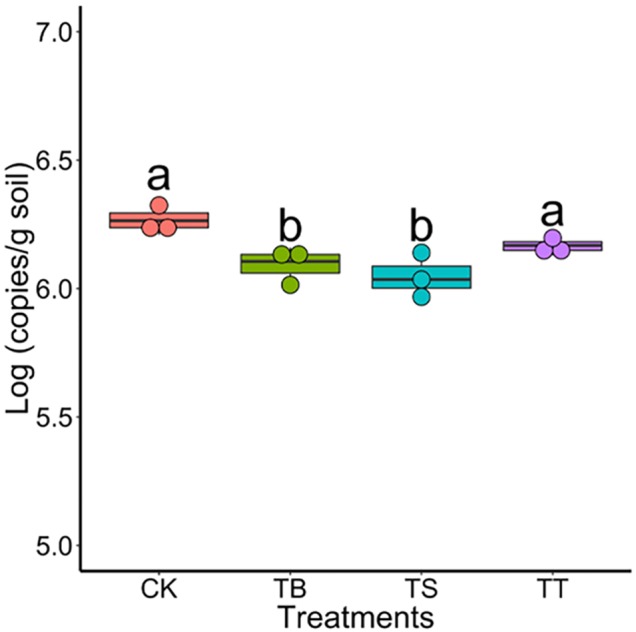
Population of *Ilyonectria* in the rhizosphere soils in field trial on 40 days (CK: control; TB: S-11; TS: S6-31; and TT: S7-1).

To study the role of antagonists in area suffering rusty disease, biocontrol studies were conducted in a field severely impacted by rusty disease. Results showed that the numbers of S-11, S6-31, and S7-1 in rhizosphere soil were 4.47 × 10^4^ (copies/g soil), 4.19 × 10^4^ (copies/g soil), 4.89 × 10^4^ (copies/g soil) on day 40, respectively. The inoculation of antagonists inhibited *Ilyonectria* in soil, especially in treatments TB and TS (Figure 6).

### Changes of Microbial Community Structure in Response to Antagonists Inoculation

**FIGURE 7:**
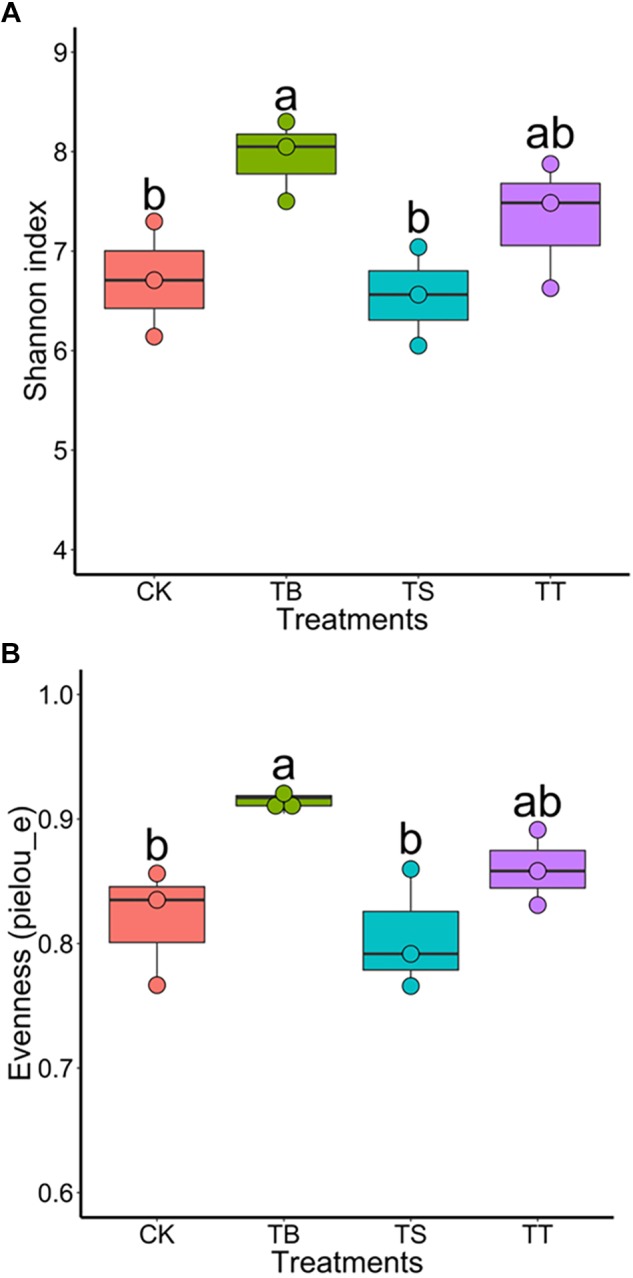
Boxplot of alpha-diversity indices for 16S rRNA gene sequences of different inoculated treatments in filed trial: **(A)** Shannon index; **(B)** Evenness (pielou_e).

To further assess the effect on soil microbiota after inoculation of antagonists in field trials, we used the same sequencing strategy described above for soil samples. The results exhibited that the treatment TB showed the highest bacterial diversity among all treatments (Figure 7A,B). For PCoA results, the inoculations became significant factors in shaping composition and structure of bacterial (*F* = 1.616, *p* = 0.001) and fungal (*F* = 1.641, *p* = 0.001) community in ginseng rhizosphere soil (Supplementary Figures S6A,B). After one-way ANOVA analysis, the result showed that all inoculated treatments had lower relative abundance of *Ilyonectria* than CK, while that of TB was the lowest (*p* < 0.05) among all treatments. Other statistically significant differences in bacterial order and fungal genera were shown in Supplementary Table S7.

## Discussion

Plants harbor a wide variety of microorganisms in both endosphere and rhizosphere. These microorganisms, including bacteria and fungi, have a strong impact on the fitness of plants (Vandenkoornhuyse et al., 2015). Most of previous studies about ginseng rusty root were based on culture-dependent methods (Reeleder and Brammall, 1994; Reeleder et al., 2006; Lu et al., 2014, 2015). However, the relationship between microbial community of ginseng and rusty disorder is still unclear. Here, we systematically researched the microbiome related to rusty roots of *P. ginseng* by metagenomic sequencing. Preliminary understanding of microbial compositions about ginseng roots were obtained.

To get insights into the microbes in rusty ginseng roots, we compared the bacteria and fungi communities between healthy and rusty periderm tissues. Our results revealed that the fungal microbiome showed severe imbalance in rusty tissues that mainly attribute to the abundance of *Ilyonectria*. *Ilyonectria*, a species complex, is commonly associated with root-rot diseases of a wide range of hosts (Cabral et al., 2011). Among the species complex, *I. mors-panacis* was genetically distinct from the other isolates and was reported as an aggressive pathogen to cause rot ginseng roots, while *I. robusta* was clustered into a different species with weak aggressiveness (Seifert et al., 2003; Cabral et al., 2011). Pathogenicity of *Ilyonectria* was also reported in many studies (Rahman and Punja, 2005; Lu et al., 2015, 2019).

The abundance of *Ilyonectria* found in endosphere showed that this genus was highly specialized to its ecological niche. Once the infection occurred, possible pathogen effectors produced by *Ilyonectria* may provide a fitness benefit to the pathogen during host colonization (Guttman et al., 2014). The invaded pathogen outcompeted other fungal genera, such as *Mortierella*, *Fusarium*, reducing the relative abundance of these genera and lowering the diversity in fungal microbiome (Figure 2A,B). Generally, most soil-borne pathogens grow saprophytically in rhizosphere so as to reach their hosts and proliferate to a certain level before infecting host tissues and escaping from rhizosphere battle zone (Berendsen et al., 2012). Compared with Hs, the Ds has an enriched *Ilyonectria*, suggesting the pathogen that infected ginseng roots originated from the rhizosphere soil.

As we know, there is a fierce battle among microorganisms in the rhizosphere and endosphere due to resource heterogeneity and availability (Raaijmakers et al., 2008; Saleem, 2015). *Fusarium*, a major fungal pathogen in ginseng, was more abundant in Hr and Hs than in Dr and Ds (Figure 2E,F and Supplementary Figure S2H). We assume this situation is attributed to the competition between *Fusarium* and *Ilyonectria* for ecological niche and nutrients. Besides, *Mortierella* was found enriched in Hr (Figure 2E,F) and its relative abundance was positively associated with P (Figure 4B). *Mortierella spp*. is ubiquitous in the bulk and rhizosphere soils (Wang et al., 2018) with an important role in keeping plant healthy by suppressing soil-borne pathogens and assisting plant with phosphorus uptake (Osorio and Habte, 2001; Miao et al., 2016). With these functions, *Mortierella* may play a key role in promoting the health of ginseng roots.

A recent study showed that infection caused by *Ilyonectria* led to the upregulation of salicylic acid (SA) in ginseng roots (Farh et al., 2019). SA, typically effective against infection caused by pathogens, is a major signaling regulator in plant (Pieterse et al., 2012). SA also influences the microbial community of roots (Lebeis et al., 2015). In our research, a decreased Shannon diversity was observed in Dr (Figure 1A). In comparison with Hr, decreased relative abundance of *Actinomycetales* and increased relative abundance of *Pseudomonadales* was observed in Dr (Figure 1E,F), suggesting that the balance between these two orders could be important to the health of ginseng roots. As one of the dominant bacterial orders, *Actinomycetales* has a powerful ecological interaction with pathogens because of its abundance in soil and various broad-spectrum antibiotics it produces (Cha et al., 2016). Herein, *Actinomycetales* with antagonistic activities could be the key to keep ginseng root free of rust. *Pseudomonadales*, another important bacterial order, is typically considered as plant growth-promoting bacteria in soil (Bertrand et al., 2001; Zamioudis et al., 2013). Besides, *Xanthomonadales*, the cause of a wide variety of serious diseases in plants (Naushad and Gupta, 2013), was enriched in Ds. Plants have been shown to adjust their root microbiome upon pathogen infection and specifically recruit a group of growth-promoting and disease resistance-inducing microbes (Berendsen et al., 2018). This could explain the enriched abundance of *Pseudomonas* in Dr and increased *Xanthomonadales* in Ds. In future, we should pay more attention to the function of these species relative to ginseng in that many species are still not classified as either pathogenic or non-pathogenic using metagenomic sequencing.

Fungicides and host resistance often cannot offer adequate and sustainable control on soil-borne diseases (Weller et al., 2002). Therefore, we isolated three antagonists with antifungal activities against *Ilyonectria*. As is known to all, *Bacillus* and *Streptomyces* are producers of antibiotics against pathogens in different plant species (Chowdhury et al., 2015; Cha et al., 2016). *Bacillus* has a good potential as a microbial agent for the biocontrol of the ginseng root rot caused by *Fusarium* (Song et al., 2014) and *Cylindrocarpon destructans* (Kim et al., 2017). With regard to *Trichoderma*, it has been one of the most popular genera of fungi commercially available as a plant growth promoting fungus and a biological control agent with the abilities to inhibit phytopathogens in many ways (Tahia et al., 2004; Keswani et al., 2014). In field trials, the relative abundance and copies of *Ilyonectria* in rhizosphere soil obviously decreased in TB after the inoculation of S-11. This result might be explained by the presence of S-11 and the remarkable raising in bacterial diversity, as bacteria could protect plants against root-derived fungi considering the negative interactions between them (Durán et al., 2018). The reduction of *Ilyonectria* may help ginseng suffering from rusty diseases recovery in the long term.

In field trials, S7-1 did not perform as well as it did in pot conditions. Differences in biocontrol abilities between pot and field trials maybe due to the differential climate conditions and soil properties. We speculated that S7-1 could protect ginseng roots away from the rusty disease at the early stage, while S-11 might be the best choice in severe rusty areas. These isolates are potential biocontrol candidates for the rusty disease. Further, disease suppressiveness is generally attributed to microbial consortia rather than to one microbial species only. Hence, application of synthetic communities has been suggested as an alternative to improve the consistency of pathogen control (Grosskopf and Soyer, 2014). In addition, RDA analysis showed that *Ilyonectria* presented different relationships with different soil properties with both positive and negative links. Therefore, changing physical and chemical properties of soil to shift the structure of microbiome and reduce the abundance of *Ilyonectria* for disease management will be a promising strategy (Vida et al., 2016). In the future, more investigation is needed to enhance our understanding of the relationship between ginseng roots and root-associate microbiome to improve ginseng yields and increase resilience toward biotic and abiotic stresses (Haney and Ausubel, 2015; Saleem et al., 2018).

## Conclusion

In conclusion, utilizing metagenomic tool, we uncovered the differences of microbial community between rusty and healthy *P. ginseng* roots. Microbial diversity is higher in healthy tissues than in diseased ginseng root tissues. Moreover, the structure of root microbiome demonstrates that the genus of *Ilyonectria* is the main microorganism that causes rusty ginseng roots. Several other bacterial and fungal taxa are also differentially distributed in healthy and diseased groups. Such differences play an important role in keeping ginseng root healthy. At last, we developed a biocontrol practice to reduce the amount of *Ilyonectria* in soil and studied the microbiota changes after treatment. This offers promising solutions to the biocontrol of rusty disease.

## Data Availability

No datasets were generated or analyzed for this study.

## Author Contributions

HM and DL designed the research. DL performed the experiments and analyzed the data. DL and HS wrote the manuscript. All authors critically revised the manuscript and approved the final version.

## Conflict of Interest Statement

The authors declare that the research was conducted in the absence of any commercial or financial relationships that could be construed as a potential conflict of interest.

## Footnotes

1 https://unite.ut.ee

2 https://www.ncbi.nlm.nih.gov/
