# Supplementary material for: Deciphering Microbiome Related to Rusty Roots of Panax ginseng and Evaluation of Antagonists Against Pathogenic Ilyonectria
Source: Front Microbiol. 2019 Jun 18;10:1350. doi: 10.3389/fmicb.2019.01350 (PMC6591430; doi:10.3389/fmicb.2019.01350)
Supplement: Supplementary file 1 [file Data_Sheet_1.docx]

Supplementary Material

**Supplementary Figures**

**
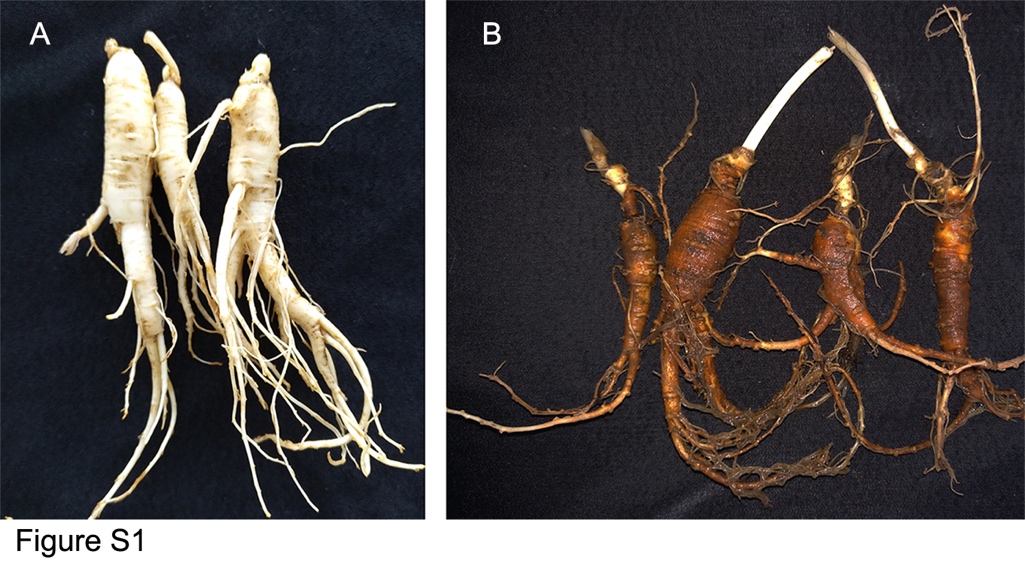
**

**Figure S1** Typical symptoms of rusty root of ginseng: (A) healthy roots (B) rusty roots.

**
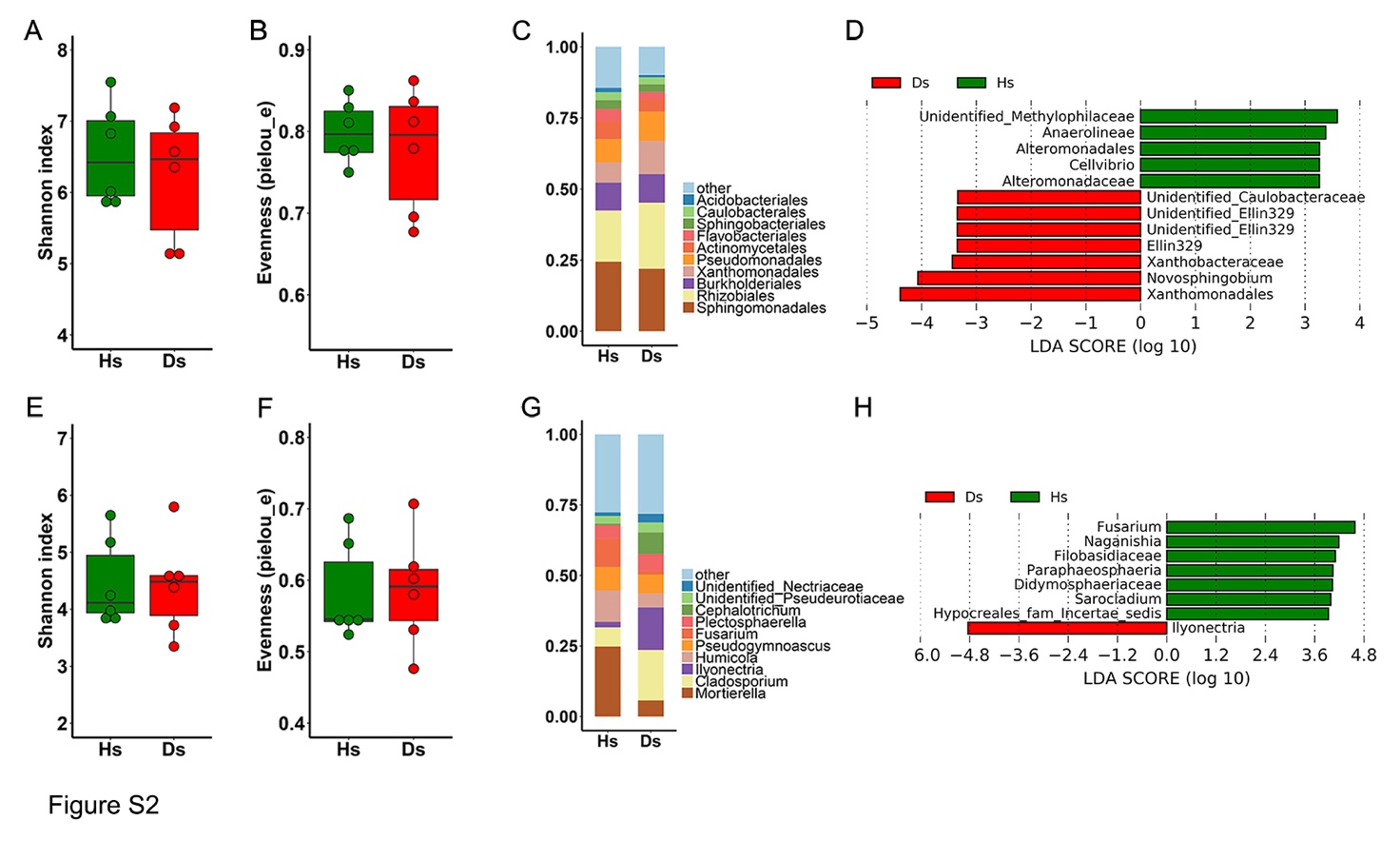
**

**Figure S2** Bacterial and fungal microbiota composition of Healthy-soil (Hs) and Diseased-soil (Ds). Boxplot of alpha-diversity indices for 16S rRNA of Hs and Ds: **(A)** Shannon index; **(B)** Evenness (pielou_e). **(C)** Relative abundances of bacterial orders in Hs and Ds. **(D)** LDA scores of the differentially abundant taxa. Taxa enriched in bacterial microbiota from Hs or Ds are indicated with a positive or negative LDA score, respectively (taxa with relative abundance > 0.001 and LDA score > 2 are shown). Boxplot of alpha-diversity indices for ITS of Hs and Ds: **(E)** Shannon index; **(F)** Evenness (pielou_e). **(G)** Relative abundances of fungal genera in Hs and Ds. **(H)** LDA scores of the differentially abundant taxa. Taxa enriched in fungal microbiota from Healthy-soil (Hs) or Diseased-soil (Ds) are indicated with a positive or negative LDA score, respectively (taxa with relative abundance > 0.001 and LDA score > 2 are shown).


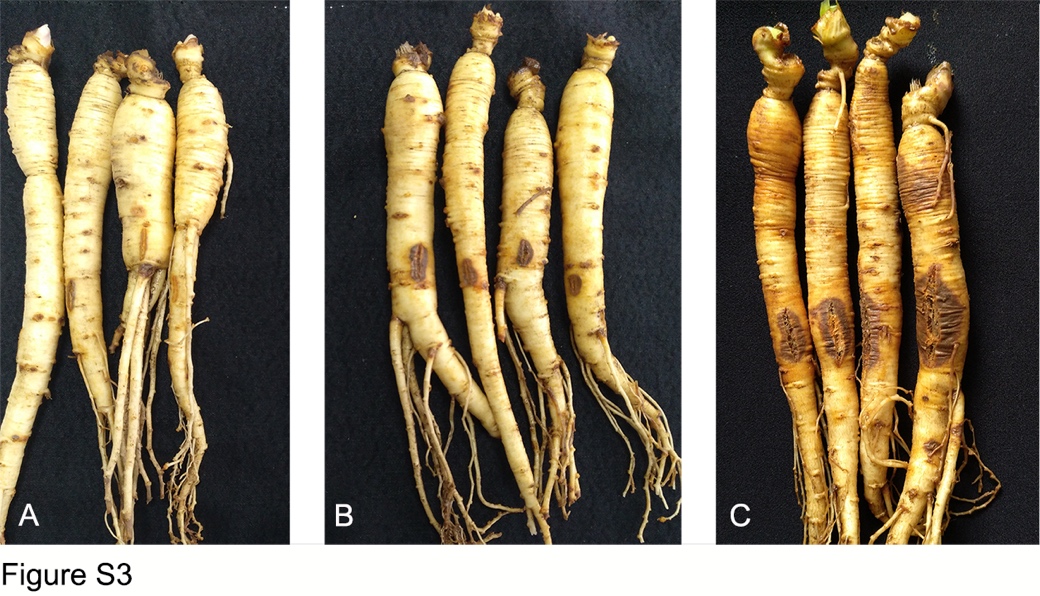


**Figure S3** Symptoms observed on ginseng roots inoculated with *Ilyonectria*. **(A)** Bare-root inoculation with sterile water, **(B)** *I. robusta* 4D-1 and **(C)** *I. mors-panacis* TH5.

**
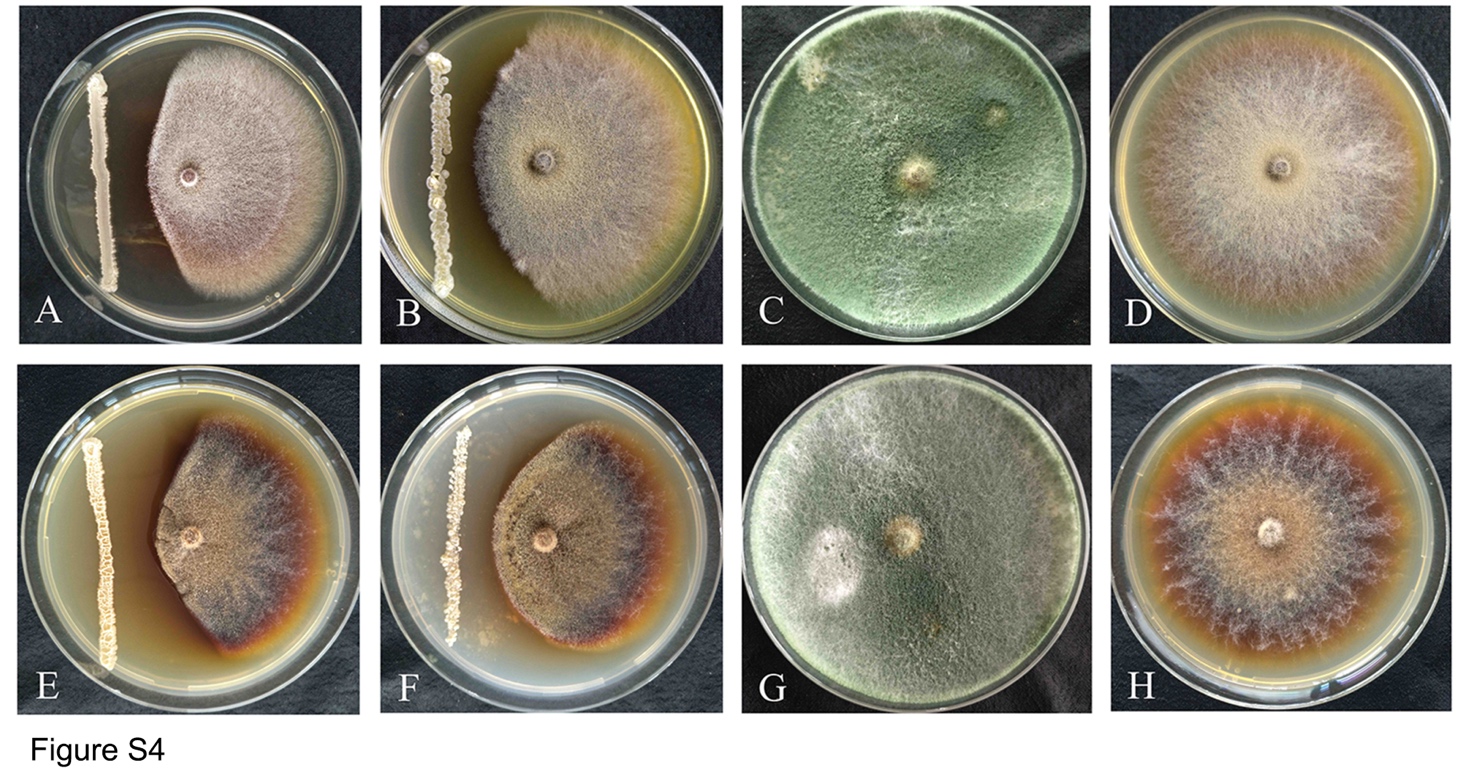
**

**Figure S4** Antagonistic activity against *I. robusta* 4D-1 and *I. mors-panacis* TH5: **(A)**: S-11 vs. 4D-1; **(B)**: S6-31 vs. 4D-1; **(C)**: S7-1 vs. 4D-1; **(D)**: control 4D-1; **(E)**: S-11 vs. TH5; **(F)**: S6-31 vs. TH5; **(G)**: S7-1 vs. TH5; **(H)**: control TH5.

**
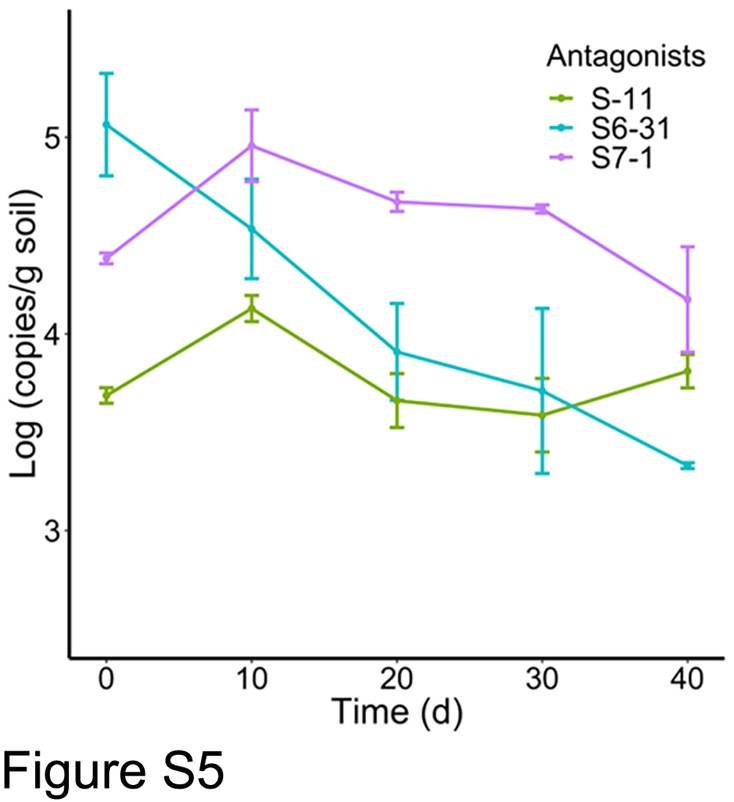
**

**Figure S5** Population dynamics of antagonists in rhizosphere soil under pot trial.

**
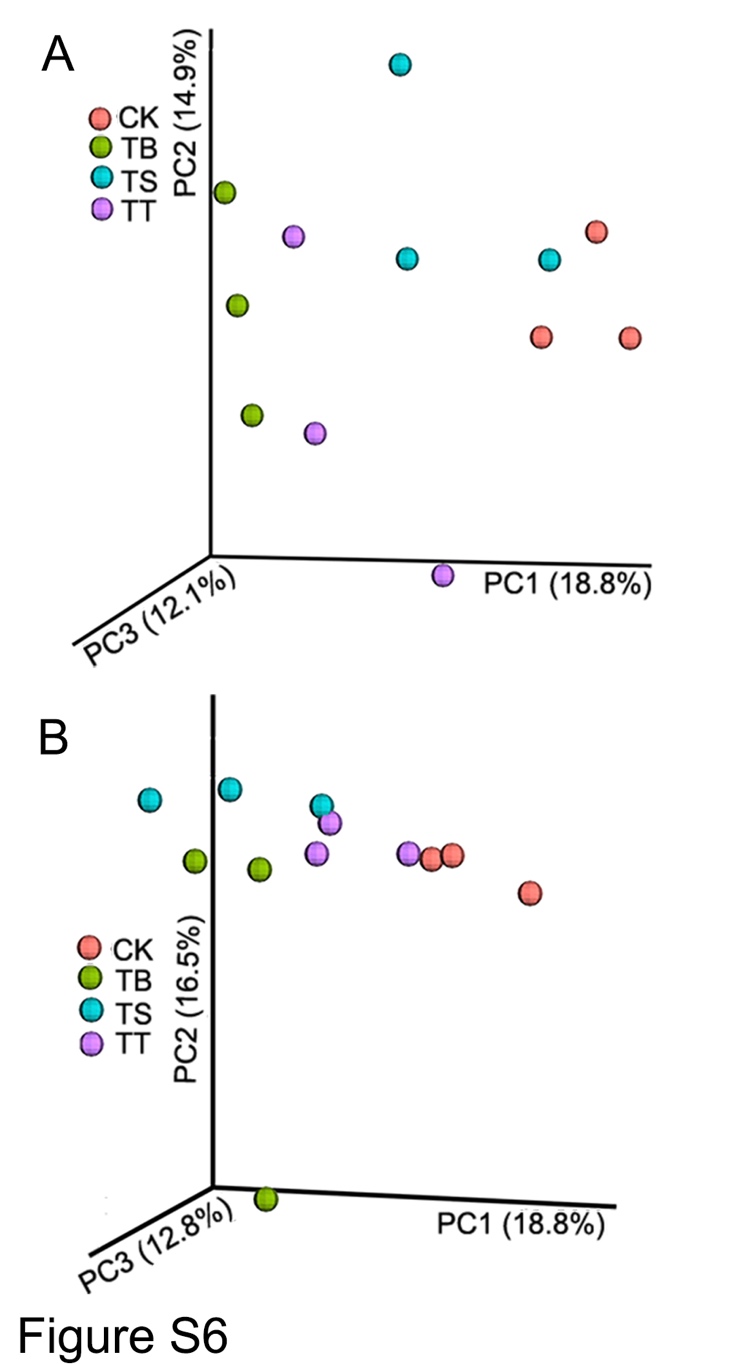
**

**Figure S6** Principal coordinate analysis (PCoA) of Bray-Curtis distances of the bacterial **(A)** and fungal communities **(B)** in different inoculated treatments in filed trial.
